# Comparative evaluation of thick and thin gingiva for dental implant loading 

**DOI:** 10.6026/973206300220077

**Published:** 2026-01-31

**Authors:** Ankit Mahajan, Kanwarjit Singh Asi, Ajay Mahajan

**Affiliations:** 1 Department of Dentistry, Government Medical College and Hospital, Kathua, Jammu & Kashmir, India; 2 Department of Periodontology and Implantology, H.P. Government Dental College Shimla, Himachal Pradesh, India; 3 Department of Periodontology and Implantology, H.P. Govt. Dental College Shimla, Himachal Pradesh, India

**Keywords:** Alveolar bone level, gingival biotype, Implant outcomes, plaque accumulation, probing depth

## Abstract

The influence of gingival biotype on the clinical and radiographic outcomes of dental implants, particularly in terms of inflammation, bone stability and overall implant success is relevant. Therefore, it is of interest to evaluate the impact of gingival biotype on the clinical and radiographic outcomes of dental implants. Patients with thick and thin gingival biotypes were assessed for parameters such as bleeding on probing, plaque accumulation, probing depth, gingival margin levels and bone loss at baseline, 3 months and 6 months. Data shows that thick gingival biotype had more favorable outcomes in terms of lower inflammation and better bone stability. Thus, we show the significant impact of gingival biotype on the clinical and radiographic outcomes of dental implants, highlighting the need for personalized treatment planning based on gingival characteristics.

## Background:

Gingival biotype refers to the thickness and contour of the gingival tissue surrounding the teeth and implants. It is categorized primarily into two types: thick and thin biotypes. The gingival biotype is a critical factor influencing the success of dental implants, as it affects both the aesthetic and functional outcomes [1]. Thick gingival biotype is characterized by dense, fibrous tissue and broad keratinized gingiva, while thin gingival biotype tends to be more delicate and fragile, with a narrower zone of keratinized tissue [2]. These differences in gingival architecture can significantly impact the way the soft and hard tissues around implants heal and respond to external factors [3]. Several studies have shown that a thicker gingiva may provide better protection against periodontal disease and improve the stability of the peri-implant tissues. This biotype has been associated with better clinical outcomes, such as reduced risk of gingival recession and greater resistance to trauma [4]. On the other hand, thin gingival biotype is more prone to soft tissue recession and bone loss around implants, leading to complications like esthetic concerns and implant failure [5]. In dental implantology, the quality and thickness of the gingiva play a key role in determining the long-term success of implant placement. Research has indicated that patients with a thick gingival biotype are less likely to experience soft tissue shrinkage, while those with a thin biotype often face more challenges regarding soft tissue stability and aesthetic outcomes. Thin gingiva is more susceptible to damage from mechanical stresses, surgical procedures and inflammation, making it more vulnerable to recession and peri-implantitis [6, 7]. The gingival biotype also influences implant design, treatment planning and surgical technique. Therefore, evaluating the gingival biotype before implant placement allows clinicians to predict potential complications and outcomes more accurately [8]. A thorough understanding of the gingival biotype's effect on implant success is essential for planning optimal treatment strategies, ensuring better esthetic results and improving patient satisfaction [9]. Therefore, it is of interest to determine the impact of gingival biotype on the clinical and radiographic changes observed around dental implants.

## Methodology:

The study was conducted at the Department of Periodontology and Implantology, Himachal Pradesh Government Dental College and Hospital, Shimla. Ethical approval for the study was obtained from the Protocol Clearance Committee and informed consent was acquired from all participants. The study aimed to evaluate the clinical and radiographic changes around dental implants placed in patients with thick and thin gingival biotypes. A total of 28 patients were enrolled in the study, all of whom were between 18 and 60 years of age. The patients were divided into two groups based on the gingival biotype: 21 patients with thick gingiva and 7 patients with thin gingiva. The participants were selected based on specific inclusion and exclusion criteria. Patients who were willing to participate, had partially dentate conditions requiring dental implants, sufficient bone and keratinized tissue for implant placement and were periodontally healthy were included. Exclusion criteria included conditions such as heart disease, previous implant placement, smoking, alcohol or drug abuse, poor oral hygiene, uncontrolled diabetes, pregnancy and active infection in the implant area. Gingival biotype was evaluated using multiple methods to determine its thickness and general appearance. The digital Vernier caliper method was employed where the gingiva was anesthetized using a topical anesthetic gel. A digital caliper was used to measure the gingival thickness at the point where the gingival margin meets the mucogingival junction in a perpendicular direction. The measurement was recorded in millimeters. In addition, visual examination of the gingiva was performed, where thick gingiva was characterized by dense and fibrotic tissue and thin gingiva appeared delicate, friable and almost translucent. The probe transparency method was also used, wherein a periodontal probe was inserted into the sulcus on the mid-facial aspect of the tooth. If the probe was visible through the gingival tissue, it was classified as thin and if not, it was considered thick. Surgical procedures were carried out following standard clinical guidelines for dental implant placement. Initially, a pre-surgical assessment was performed, which included a complete medical, dental and periodontal assessment, including plaque index, gingival index and vital signs. Radiographic analysis was done with intraoral periapical radiographs (IOPA), orthopantomograms (OPG) and a CBCT scan. After the initial assessment, surgical stents were fabricated using preoperative impressions and diagnostic wax-ups. The implant sites were assessed for bone height and width using CBCT and implant sizes were selected accordingly. During the implant placement procedure, a crestal incision was made to expose the implant site. The surgical stent was placed over the crest to mark the position and a pilot drill was used to initiate the osteotomy. Subsequent drills of increasing diameter were used to prepare the final implant site. After implant placement, periapical radiographs were taken to confirm the position of the implant. The second stage surgery involved uncovering the implant cover screw and placing a gingival former to shape the surrounding tissue. After osseointegration, final restoration was completed by taking final impressions with silicone material to fabricate the implant-supported superstructure. The prosthesis was placed and the implant position was verified using periapical radiographs. The clinical parameters were assessed at baseline, 3 months and 6 months after implant placement. These included bleeding on probing (BOP), which was evaluated using the modified sulcus bleeding index (mGI); plaque accumulation, measured using the modified plaque index (mPI); probing depth (PD), which was measured at mesial, distal, lingual and buccal sites with a plastic probe; and gingival margin level (GML), measured from the gingival margin to the implant shoulder. Implant mobility was assessed using a clinical scale to determine any signs of instability. The interdental papillary volume index (IDPVI), evaluated using the Jemt index was used to assess the presence and volume of the interdental papilla. Alveolar bone levels were monitored using radiographs at mesial and distal aspects around the implant. Additionally, Visual Analog Scale (VAS) was used to assess patient satisfaction and the esthetic outcomes of the treatment. Patients were followed up at 3 months and 6 months after functional loading to assess clinical parameters, radiographic changes and overall implant success. This methodology provided a comprehensive evaluation of the effect of gingival biotype on dental implant outcomes, offering essential data for improving treatment planning and predicting long-term success.

## Results:

**Figure 1:**
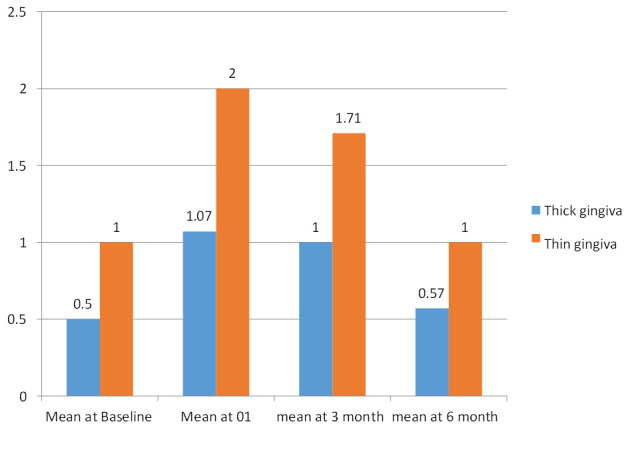
Comparative evaluation of bleeding on probing at various intervals in thick vs thin gingival biotype

**Figure 2:**
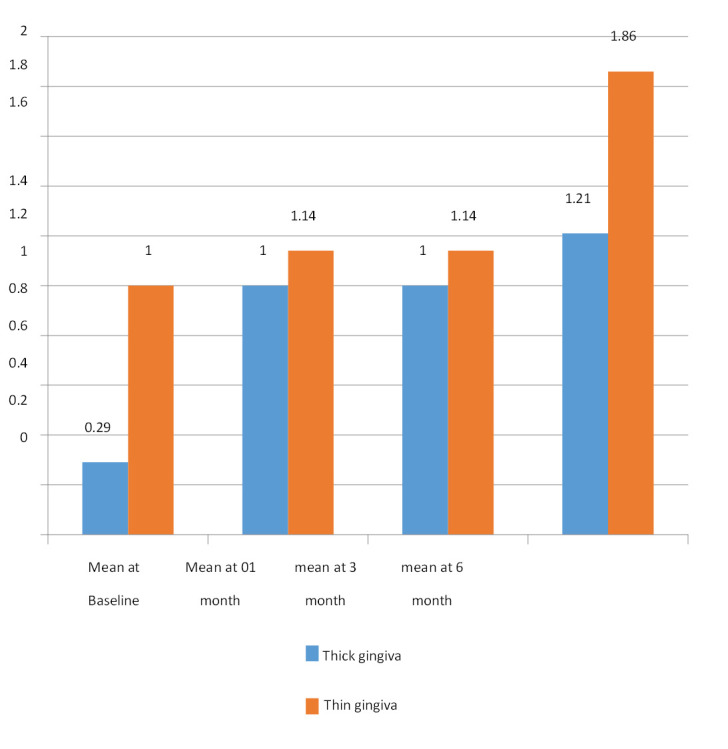
Comparative evaluation of plaque level at various intervals in thick vs thin gingival biotype

Baseline values for bleeding on probing in thin gingival biotype were 1.00±0.000 and in thick gingival biotype was 0.50± 0.51. At 3 months the mean bleeding on probing for thin gingival biotype was 1.71± 0.488. In thick gingival biotype the mean value was 1.00±0.000. At 6 months the mean bleeding on probing for thin gingival biotype was 1.000±0.000. In thick gingival biotype the mean value was 0.57±0.514. Negligible difference was found between thick and thin gingival biotypes when intergroup comparison was done based on bleeding on probing (Table 1, Figure 1). At baseline the mean PI in thin gingival biotype was 1.00±0.00 and in thick gingival biotype the value was 0.29±0.469. At 3rd month, mean PI in thin gingival biotype was 1.14±0.378 and in thick gingival biotype was 1.00±0.00. At 6th month following implant placement, the mean PI in thin gingival biotype was 1.86±0.378 thick gingival biotype was 1.21±0.426. A significant difference was seen in the values of Mean plaque index within the two gingival biotypes (Table 2). Comparative evaluate of plaque level at various intervals in thick versus thin gingival biotype. This figure shows the differences in plaque index between the two gingival biotypes over time at baseline, one month, three months and six months (Figure 2). Intergroup comparison of probing depth at different intervals of time. The table compares the mean probing depths in thin and thick gingival biotypes at baseline, three months and six months for both mesial and distal sites. Statistical significance was observed at different time intervals (Table 3).

## Probing depth (PD) for mesial side:

**Figure 3:**
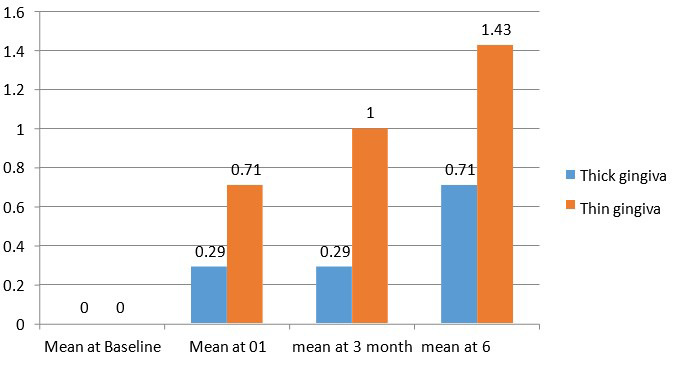
Comparative evaluation of implant mobility at different intervals in thick vs thin gingival biotype

Baseline values for mean probing depth in thin gingival biotype were 3.00±0.00 and in thick gingival biotype, the mean value was 2.29±0.469. At 3 months, in thin gingival biotype the mean value was 4.00±0.00 and thick gingival biotype the value was 3.00±0.00. At 6 months, the mean value in the thin gingival biotype was 4.00±0.00 and in the thick gingival biotype, the value was 3.43±0.51. Probing depth (PD) for the distal side. The Mean probing depth in the thin gingival biotype at baseline was 3.00±0.00 and in the thick gingival biotype, it was 2.29±0.469. At 3 months, in thin gingival biotype the mean value was 4.00±0.00 and thick gingival biotype the value was 3.00±0.00. At 6 months in the thin gingival biotype, the mean value was 4.00±0.00 and in the thick gingival biotype, the value was 3.00±0.00. A significant difference was seen in the mean probing depth at different intervals between two gingival biotypes. Gingival margin levels at baseline on mesial side were studied in the two gingival biotypes. In thin gingival biotype the mean value of GML was 3.00±0.00 and in thick gingival biotype the mean value of GML was found to be 2.36±0.49. At 3 months the mean GML in thin gingival biotype was 3.57±0.535 and in thick gingival biotype the value was 2.43±0.514. At 6 months the mean GML in thin biotype was 4.00±0.00 and in thick gingival biotype the mean value was 3.00±0.00. Gingival margin levels at baseline on distal side were studied in the two gingival biotypes. In thin gingival biotype GML was 2.86±0.378 and in thick gingival biotype the mean value of GML was found to be 2.00±0.00. At 3 months, the mean value of GML in the thin gingival biotype was 4.00±0.00 and in the thick gingival biotype, the mean value of GML was 3.00±0.00. At 6 months, the mean GML value in the thin biotype was 4.00±0.00 and in the thick gingival biotype, the mean value was 3.57±0.514 (Table 4). Both gingival biotypes showed a significant mean difference in the gingival margin level. No significant difference was found between both gingival biotypes when Implant mobility was assessed at different intervals time (Table 5, Figure 3). At baseline the mean value of alveolar bone levels for both gingival biotypes was 0.00±0.00. At 3 months the mean value of alveolar bone levels for thin gingival biotype was 0.44±0.34 and for thick gingival biotype the value was 0.329±0.046. At 6 months the mean value alveolar bone levels for thin gingival biotype was 0.493±0.034 and for thick gingival biotype mean value 0.375±0.0427.

## Discussion:

This study aimed to evaluate the impact of gingival biotype on the clinical and radiographic outcomes of dental implants. The results indicated that thick gingival biotype exhibited more favorable outcomes compared to thin gingival biotype, aligning with findings from previous research. A systematic review by da Silva *et al.* (2025) [4] reported that both thin and thick gingival phenotypes had high implant survival rates (>91%) over a 5-year period. However, thin phenotypes were associated with a higher risk of peri-implantitis, leading to marginal bone loss beyond expected levels. This suggests that while both biotypes can achieve high survival rates, thin biotypes may be more susceptible to complications affecting marginal bone levels. In our study, thin gingival biotype demonstrated higher bleeding on probing, plaque accumulation, probing depth and gingival margin level changes, which are indicative of increased susceptibility to inflammation and recession. These findings are consistent with those of Parihar *et al.* (2025) [10], who observed that thin biotypes exhibited greater susceptibility to gingival recession and mucosal level changes over time, even with platform-switched implants and immediate provisionalization. Furthermore, the study by Sharma *et al.* (2024) [11] highlighted that thin gingival biotype is more prone to interdental tissue destruction, as it is associated with a papilla of lesser dimension than that of thick gingival biotype. This aligns with our observation of reduced interdental papillary volume in the thin biotype group. The findings from this study underscore the importance of assessing gingival biotype during treatment planning for dental implants. Recognizing the differences in gingival biotypes can guide clinicians in predicting potential complications and tailoring surgical and prosthetic approaches to enhance the long-term success of implant therapy.

## Conclusion:

While both thin and thick gingival biotypes can achieve successful implant outcomes, thin biotypes may require more meticulous planning and management to mitigate the risk of peri-implant complications. Further research with larger sample sizes and longer follow-up periods is warranted to deepen our understanding of the influence of gingival biotype on implant success.

## Figures and Tables

**Figure 4:**
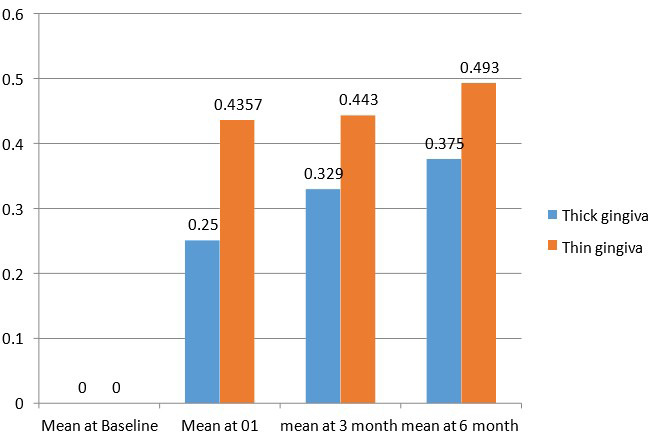
Comparative evaluation of alveolar bone level at various intervals in thick vs thin gingival biotype Alveolar bone level changes

**Figure 5:**
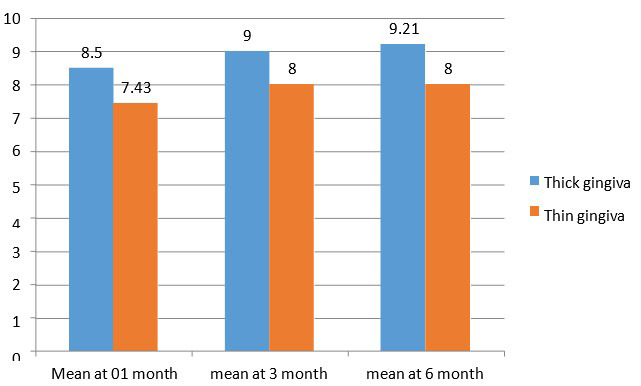
Comparative evaluation of visual analogue scale in thick versus thin gingival biotype at various intervals

**Table 1:** Intergroup comparison of bleeding on probing at different intervals acc. to the thickness of the Gingiva

**TIME INTERVAL**	**Group**	**N**	**Mean**	**Std. Deviation**	**Std. Error Mean**	**Mean diff**	**T value**	**P value**
Baseline	Thick Gingiva	14	0.5	0.519	0.139			
	Thin Gingiva	7	1	0	0	-0.5	-3.606	0.003*
At one month	Thick Gingiva	14	1.07	0.267	0.071			
	Thin Gingiva	7	2	0	0	-0.929	-13	0.000*
At three months	Thick Gingiva	14	1	0	0			
	Thin Gingiva	7	1.71	0.488	0.184	-0.714	-5.627	0.000*
At six months	Thick Gingiva	14	0.57	0.514	0.137			
	Thin Gingiva	7	1	0	0	-0.429	-3.122	0.008*

**Table 2:** Intergroup comparison of plaque accumulation at different intervals according to thickness of Gingiva

**Duration.**	**Group**	**/N**	**Mean**	**Std. Deviation**	**Std. Error Mean**	**Mean diff**	**T value**	**P value**
Baseline	Thick Gingiva	14	0.29	0.469	0.125			
	Thin Gingiva	7	1	0	0	-0.714	-5.7	0.000*
At one month	Thick Gingiva	14	1	0	0			
	Thin Gingiva	7	1.14	0.378	0.143	-0.143	-1	0.3**
At three months	Thick Gingiva	14	1	0	0			
	Thin Gingiva	7	1.14	0.378	0.143	-0.143	-1.45	0.1**
At six months	Thick Gingiva	14	1.21	0.426	0.114	-0.643	-3.38	0.003*

**Table 3:** Intergroup comparison of probing depth at different intervals of time

**SITE**	**Group**	**N**	**Mean**	**Std.Deviation**	**Std. Error Mean**	**Mean diff**	**T value**	**P value**
Mesial at baseline	Thick Gingiva	14	2.29	0.469	0.125			
	Thin Gingiva	7	3	0	0	-0.714	-5.7	0.000*
Distal at baseline	Thick Gingiva	14	2.29	0.469	0.125			
	Thin Gingiva	7	3	0	0			
	Thin Gingiva	7	2.86	0.378	0.143			
	Thin Gingiva	7	3.14	0.378	0.143	-0.714	-5.7	0000*
Mesial at three month	Thick Gingiva	14	3	.00^a^	0	-------	-------	
	Thin Gingiva	7	4	.00^a^	0			-
Distal at three month	Thick Gingiva	14	3.07	0.267	0.071			
	Thin Gingiva	7	3.57	0.535	0.202			
	Thin Gingiva	7	3.43	0.535	0.202	-0.5		0.001*
Mesial at six months	Thick Gingiva	14	3.43	0.514	0.137			
	Thin Gingiva	7	4	0	0	-0.571	-2.9	0.000*
Distal at six months	Thick Gingiva	14	3	.00^a^	0			
	Thin Gingiva	7	4	.00^a^	0			---------
	Thin Gingiva	7	3.86	0.378	0.143	-------	-------	-

**Table 4:** Intergroup comparison of the gingival marginal level (GML) at different intervals according to the thickness of the Gingiva

**SITE.**	**Group**	**N**	**Mean**	**Std.Deviation**	**Std. Error Mean**	**Mean diff**	**T value**	**P value**
Mesial at baseline	Thick Gingiva	14	2.36	0.497	0.133			
	Thin Gingiva	7	3	0	0	-0.643	-4.837	.000*
	Thin Gingiva	7	3	0	0			
Distal at baseline	Thick Gingiva	14	2	0	0			
	Thin Gingiva	7	2.86	0.378	0.143	-0.857	-8.718	.000*
	Thin Gingiva	7	3.86	0.378	0.143			
Mesial at three months	Thick Gingiva	14	2.43	0.514	0.137			
	Thin Gingiva	7	3.57	0.535	0.202	-1.143	-4.745	.000*
Distal at three months	Thick Gingiva	14	3	.000^a^	0			
	Thin Gingiva	7	4	.00^a^	0	--------	---------	---------
	Thin Gingiva	7	3.86	0.378	0.143	-	----	----
Mesial at six months	Thick Gingiva	14	3.5	0.519	0.139			
	Thin Gingiva	7	4	0	0	-0.5	-3.606	.003*
Distal at six months	Thick Gingiva	14	3.57	0.514	0.137			
	Thin Gingiva	7	4	0	0	-0.429	-2.179	.04*
	Thin Gingiva	7	4	.00^a^	0			

**Table 5:** Intergroup comparison of implant mobility at different intervals thickness of Gingiva

	**Group**	**N**	**Mean**	**Std. Deviation**	**Std. Error Mean**	**Mean diff**	**T value**	**P value**
Baseline	Thick Gingiva	14	0	.00^a^	0	--------	---------	---------
	Thin Gingiva	7	0	.00^a^	0	---	----	----
At three months	Thick Gingiva	14	0.29	0.469	0.125			
	Thin Gingiva	7	1	0	0	-0.286	-1.221	0.237
At six months	Thick Gingiva	14	0.71	0.469	0.125	0.143	1.472	0.165
	Thin Gingiva	7	1.43	0.535	0.202	0.357	1.806	0.109

**Table 6:** Intergroup comparison of interdental papillary volume at various time intervals according to the thickness of the gingiva

**SITE**	**Group**	**N**	**Mean**	**Std. Deviation**	**Std. Error Mean**	**Mean diff**	**T value**	**P value**
Mesial at baseline	Thick Gingiva	14	2.93	0.267	0.071			
	Thin Gingiva	7	2.71	0.488	0.184	0.214	-4.837	0.01*
Distal at baseline	Thick Gingiva	14	2.43	0.514	0.137			
	Thin Gingiva	7	2.71	0.488	0.184			
	Thin Gingiva	7	2	0	0			
	Thin Gingiva	7	1.71	0.488	0.184	-0.286	-2.687	0.1**
Mesial at three month	Thick Gingiva	14	2.07	0.475	0.127			
	Thin Gingiva	7	2	0	0	0.071	-8.718	.000*
Distal at three baseline	Thick Gingiva	14	2.21	0.426	0.114			
	Thin Gingiva	7	1.14	0.378	0.143	1.071	-4.745	.007*
Mesial at six months	Thick Gingiva	14	2.21	0.579	0.155			
	Thin Gingiva	7	1.43	0.535	0.202	0.786	-4.919	.000*
Distal at six months	Thick Gingiva	14	2.21	0.579	0.155			
	Thin Gingiva	7	1	0	0	1.214	-2.121	.003*

**Table 7:** Intergroup comparison of bone level at various intervals acc. to thickness of Gingiva

**Duration**	**Group**	**N**	**Mean**	**Std.Deviation**	**Std.Error Mean**	**Mean diff**	**T value**	**P value**
Baseline	1	14	0	.00^a^	0		------	------
	2	7	0	.00^a^	0	------	-	-
At three months	1	14	0.329	0.0469	0.0125		-6.314	
	2	7	0.443	0.0345	0.013	-0.1179		.000*
At six months	1	14	0.375	0.0427	0.0114			
	2	7	0.493	0.0345	0.013	-0.1179	-6.798	.000*
